# Inebriism

**Published:** 1885-10

**Authors:** 


Inebriism. A pathological and psychological study. By T. L. WRIGHT, M. D. Columbus, O.: William Hubbard. 1885.

Some of the views expressed in this work are extreme and are not generally accepted by the profession, but the book has its place in the current medical literature, and, as said by one reviewer, "It is a work that would make a decided impression for good upon the minds of the young, if studied as a text-book in the higher grades of public schools." For this field, it is, indeed, a most excellent work. The care and treatment of the inebriate is *the* question of the time, and this work gives some practical lessons on the subject, which are much needed. It can be obtained from the author at Bellefontaine, O. Price, $1.25. Sent free, by mail, on receipt of above amount.

